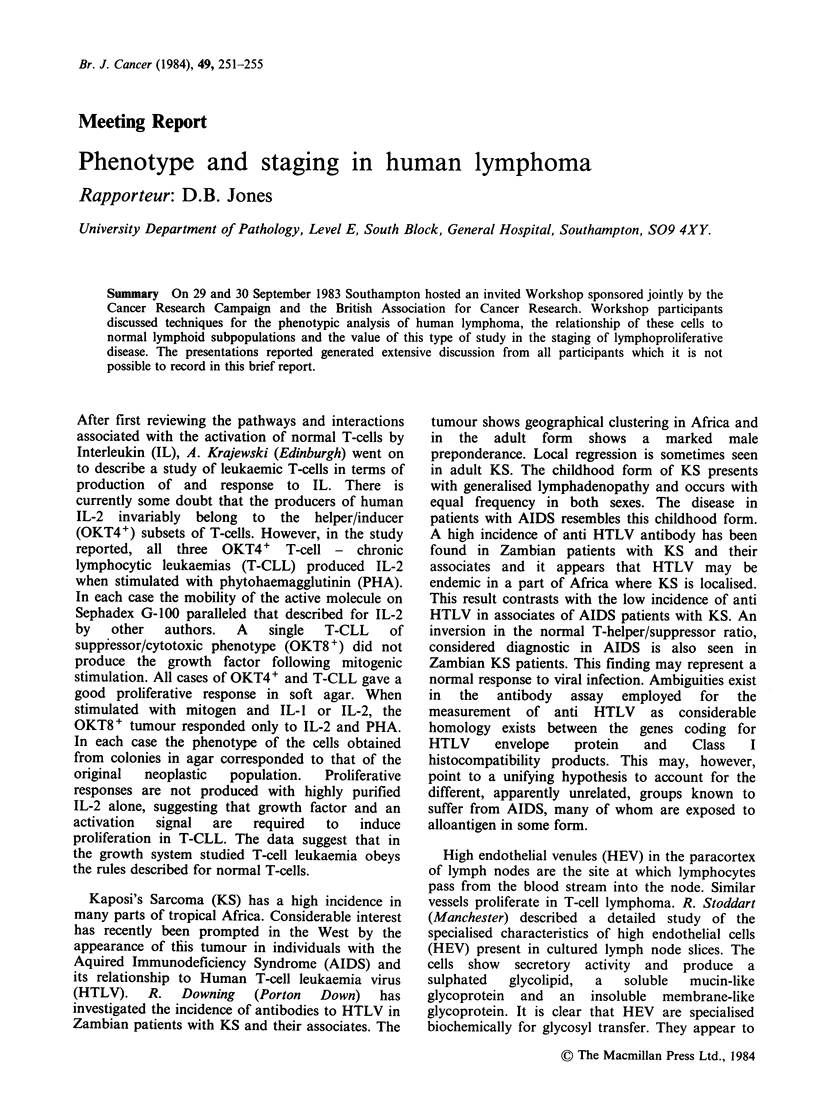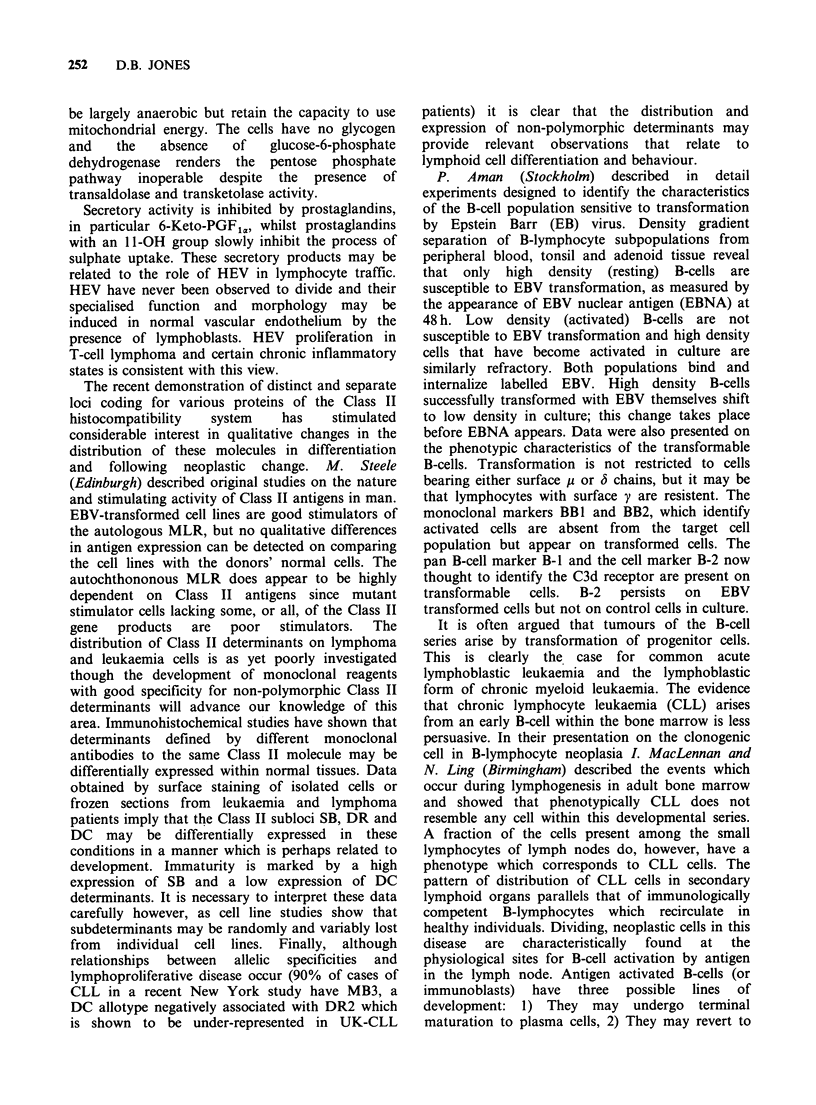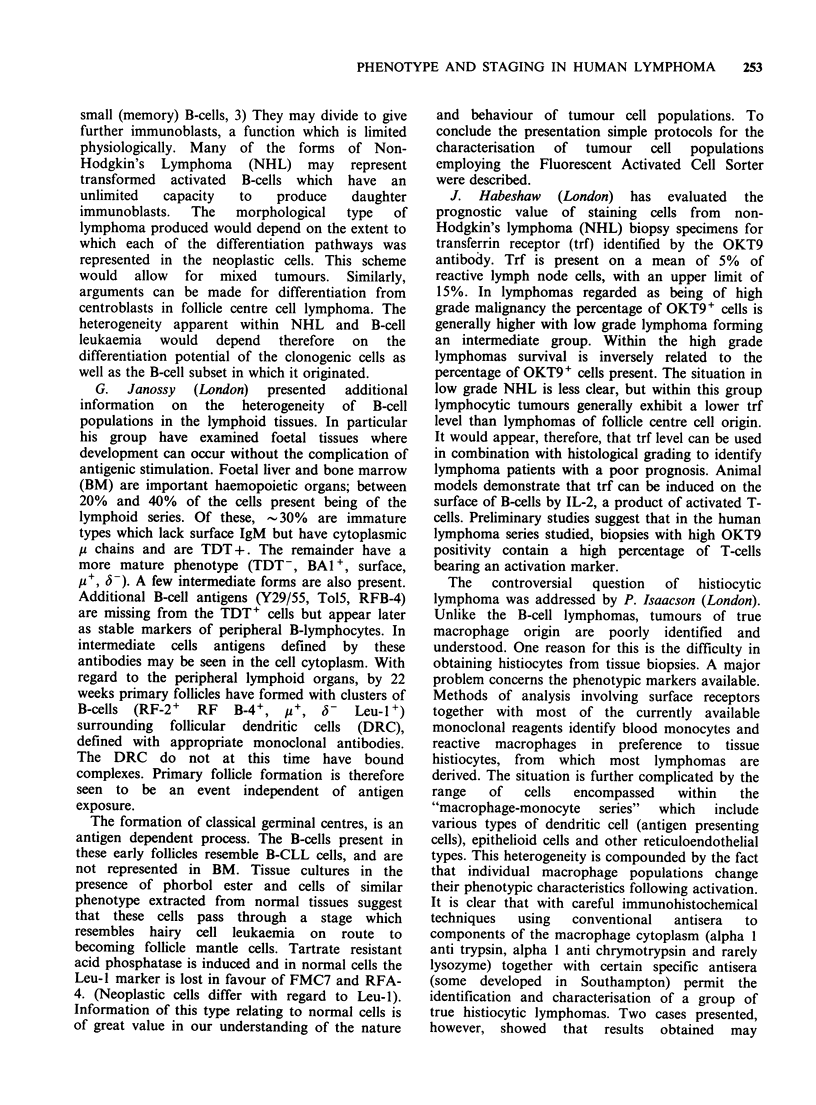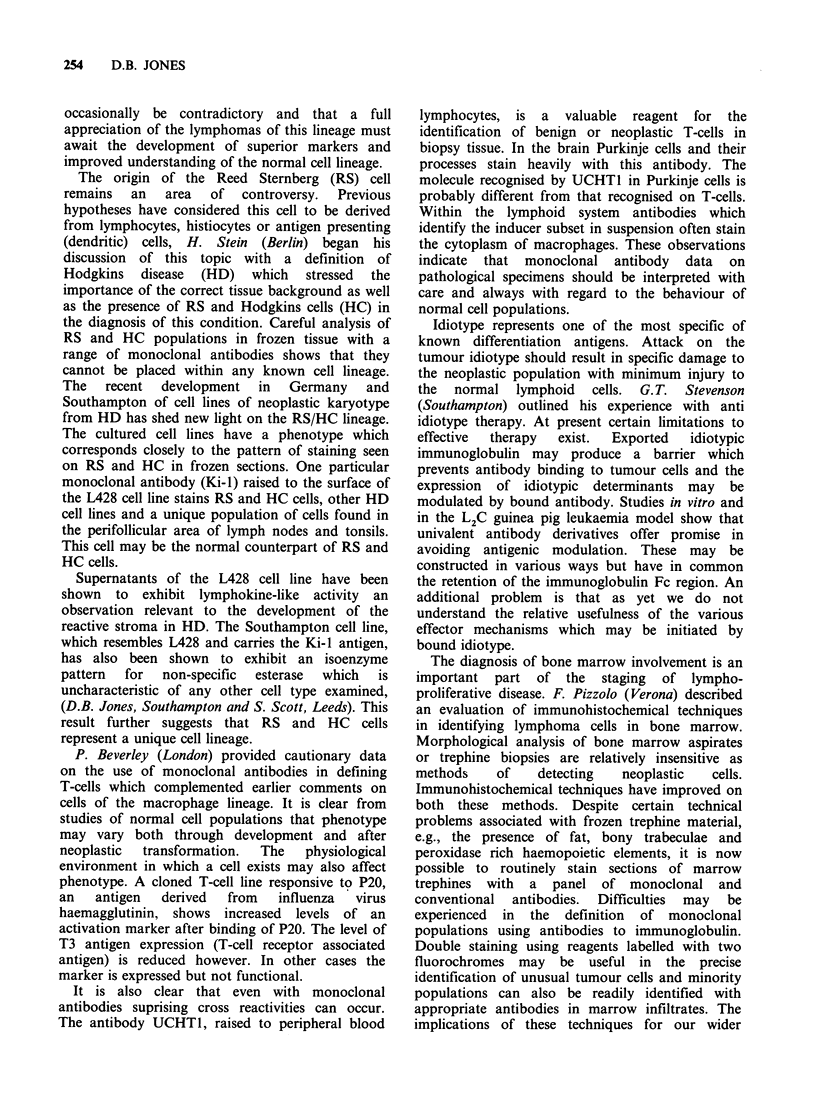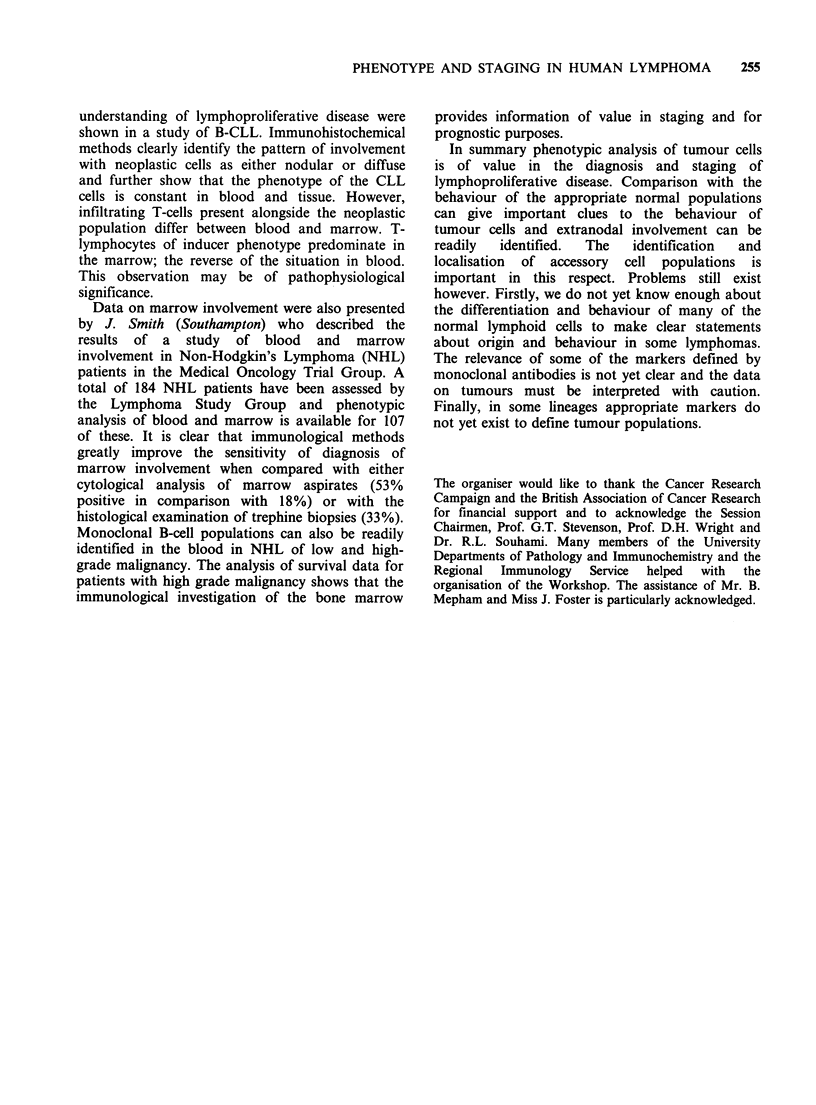# CRC/BACR Invited Workshop: Southampton, 29-30 September 1983 Phenotype Staging in Human Lymphoma

**Published:** 1984-02

**Authors:** 

## Abstract

On 29 and 30 September 1983 Southampton hosted an invited Workshop sponsored jointly by the Cancer Research Campaign and the British Association for Cancer Research. Workshop Participants discussed techniques for the phenotypic analysis of human lymphoma, the relationship of these cells to normal lymphoid subpopulations and the value of this type of study in the staging of lymphoproliferative disease. The presentations reported generated extensive discussion from all participants which it is not possible to record in this brief report.


Br. J. Cancer (1984), 49, 251–255

### Meeting Report

### Phenotype and staging in human lymphoma

*Rapporteur*: D.B. Jones

*University Department of Pathology, Level E, South Block, General Hospital, Southampton, SO9 4XY.*

**Summary** On 29 and 30 September 1983 Southampton hosted an invited Workshop sponsored jointly by the Cancer Research Campaign and the British Association for Cancer Research. Workshop participants discussed techniques for the phenotypic analysis of human lymphoma, the relationship of these cells to normal lymphoid subpopulations and the value of this type of study in the staging of lymphoproliferative disease. The presentations reported generated extensive discussion from all participants which it is not possible to record in this brief report.

After first reviewing the pathways and interactions associated with the activation of normal T-cells by Interleukin (IL), *A. Krajewski* (*Edinburgh*) went on to describe a study of leukaemic T-cells in terms of production of and response to IL. There is currently some doubt that the producers of human IL-2 invariably belong to the helper/inducer ($OKT4^+$) subsets of T-cells. However, in the study reported, all three $OKT4^+$ T-cell – chronic lymphocytic leukaemias (T-CLL) produced IL-2 when stimulated with phytohaemagglutinin (PHA). In each case the mobility of the active molecule on Sephadex G-100 paralleled that described for IL-2 by other authors. A single T-CLL of suppressor/cytotoxic phenotype ($OKT8^+$) did not produce the growth factor following mitogenic stimulation. All cases of $OKT4^+$ and T-CLL gave a good proliferative response in soft agar. When stimulated with mitogen and IL-1 or IL-2, the $OKT8^+$ tumour responded only to IL-2 and PHA. In each case the phenotype of the cells obtained from colonies in agar corresponded to that of the original neoplastic population. Proliferative responses are not produced with highly purified IL-2 alone, suggesting that growth factor and an activation signal are required to induce proliferation in T-CLL. The data suggest that in the growth system studied T-cell leukaemia obeys the rules described for normal T-cells.

Kaposi's Sarcoma (KS) has a high incidence in many parts of tropical Africa. Considerable interest has recently been prompted in the West by the appearance of this tumour in individuals with the Aquired Immunodeficiency Syndrome (AIDS) and its relationship to Human T-cell leukaemia virus (HTLV). *R. Downing* (*Porton Down*) has investigated the incidence of antibodies to HTLV in Zambian patients with KS and their associates. The tumour shows geographical clustering in Africa and in the adult form shows a marked male preponderance. Local regression is sometimes seen in adult KS. The childhood form of KS presents with generalised lymphadenopathy and occurs with equal frequency in both sexes. The disease in patients with AIDS resembles this childhood form. A high incidence of anti HTLV antibody has been found in Zambian patients with KS and their associates and it appears that HTLV may be endemic in a part of Africa where KS is localised. This result contrasts with the low incidence of anti HTLV in associates of AIDS patients with KS. An inversion in the normal T-helper/suppressor ratio, considered diagnostic in AIDS is also seen in Zambian KS patients. This finding may represent a normal response to viral infection. Ambiguities exist in the antibody assay employed for the measurement of anti HTLV as considerable homology exists between the genes coding for HTLV envelope protein and Class I histocompatibility products. This may, however, point to a unifying hypothesis to account for the different, apparently unrelated, groups known to suffer from AIDS, many of whom are exposed to alloantigen in some form.

High endothelial venules (HEV) in the paracortex of lymph nodes are the site at which lymphocytes pass from the blood stream into the node. Similar vessels proliferate in T-cell lymphoma. *R. Stoddart* (*Manchester*) described a detailed study of the specialised characteristics of high endothelial cells (HEV) present in cultured lymph node slices. The cells show secretory activity and produce a sulphated glycolipid, a soluble mucin-like glycoprotein and an insoluble membrane-like glycoprotein. It is clear that HEV are specialised biochemically for glycosyl transfer. They appear to

© The Macmillan Press Ltd., 1984

be largely anaerobic but retain the capacity to use mitochondrial energy. The cells have no glycogen and the absence of glucose-6-phosphate dehydrogenase renders the pentose phosphate pathway inoperable despite the presence of transaldolase and transketolase activity.

Secretory activity is inhibited by prostaglandins, in particular 6-Keto-$PGF_{1\alpha}$, whilst prostaglandins with an 11-OH group slowly inhibit the process of sulphate uptake. These secretory products may be related to the role of HEV in lymphocyte traffic. HEV have never been observed to divide and their specialised function and morphology may be induced in normal vascular endothelium by the presence of lymphoblasts. HEV proliferation in T-cell lymphoma and certain chronic inflammatory states is consistent with this view.

The recent demonstration of distinct and separate loci coding for various proteins of the Class II histocompatibility system has stimulated considerable interest in qualitative changes in the distribution of these molecules in differentiation and following neoplastic change. *M. Steele* (*Edinburgh*) described original studies on the nature and stimulating activity of Class II antigens in man. EBV-transformed cell lines are good stimulators of the autologous MLR, but no qualitative differences in antigen expression can be detected on comparing the cell lines with the donors' normal cells. The autochthononous MLR does appear to be highly dependent on Class II antigens since mutant stimulator cells lacking some, or all, of the Class II gene products are poor stimulators. The distribution of Class II determinants on lymphoma and leukaemia cells is as yet poorly investigated though the development of monoclonal reagents with good specificity for non-polymorphic Class II determinants will advance our knowledge of this area. Immunohistochemical studies have shown that determinants defined by different monoclonal antibodies to the same Class II molecule may be differentially expressed within normal tissues. Data obtained by surface staining of isolated cells or frozen sections from leukaemia and lymphoma patients imply that the Class II subloci SB, DR and DC may be differentially expressed in these conditions in a manner which is perhaps related to development. Immaturity is marked by a high expression of SB and a low expression of DC determinants. It is necessary to interpret these data carefully however, as cell line studies show that subdeterminants may be randomly and variably lost from individual cell lines. Finally, although relationships between allelic specificities and lymphoproliferative disease occur (90% of cases of CLL in a recent New York study have MB3, a DC allotype negatively associated with DR2 which is shown to be under-represented in UK-CLL patients) it is clear that the distribution and expression of non-polymorphic determinants may provide relevant observations that relate to lymphoid cell differentiation and behaviour.

*P. Aman* (*Stockholm*) described in detail experiments designed to identify the characteristics of the B-cell population sensitive to transformation by Epstein Barr (EB) virus. Density gradient separation of B-lymphocyte subpopulations from peripheral blood, tonsil and adenoid tissue reveal that only high density (resting) B-cells are susceptible to EBV transformation, as measured by the appearance of EBV nuclear antigen (EBNA) at 48 h. Low density (activated) B-cells are not susceptible to EBV transformation and high density cells that have become activated in culture are similarly refractory. Both populations bind and internalize labelled EBV. High density B-cells successfully transformed with EBV themselves shift to low density in culture; this change takes place before EBNA appears. Data were also presented on the phenotypic characteristics of the transformable B-cells. Transformation is not restricted to cells bearing either surface $\mu$ or $\delta$ chains, but it may be that lymphocytes with surface $\gamma$ are resistent. The monoclonal markers BB1 and BB2, which identify activated cells are absent from the target cell population but appear on transformed cells. The pan B-cell marker B-1 and the cell marker B-2 now thought to identify the C3d receptor are present on transformable cells. B-2 persists on EBV transformed cells but not on control cells in culture.

It is often argued that tumours of the B-cell series arise by transformation of progenitor cells. This is clearly the case for common acute lymphoblastic leukaemia and the lymphoblastic form of chronic myeloid leukaemia. The evidence that chronic lymphocyte leukaemia (CLL) arises from an early B-cell within the bone marrow is less persuasive. In their presentation on the clonogenic cell in B-lymphocyte neoplasia *I. MacLennan and N. Ling* (*Birmingham*) described the events which occur during lymphogenesis in adult bone marrow and showed that phenotypically CLL does not resemble any cell within this developmental series. A fraction of the cells present among the small lymphocytes of lymph nodes do, however, have a phenotype which corresponds to CLL cells. The pattern of distribution of CLL cells in secondary lymphoid organs parallels that of immunologically competent B-lymphocytes which recirculate in healthy individuals. Dividing, neoplastic cells in this disease are characteristically found at the physiological sites for B-cell activation by antigen in the lymph node. Antigen activated B-cells (or immunoblasts) have three possible lines of development: 1) They may undergo terminal maturation to plasma cells, 2) They may revert to small (memory) B-cells, 3) They may divide to give further immunoblasts, a function which is limited physiologically. Many of the forms of Non-Hodgkin's Lymphoma (NHL) may represent transformed activated B-cells which have an unlimited capacity to produce daughter immunoblasts. The morphological type of lymphoma produced would depend on the extent to which each of the differentiation pathways was represented in the neoplastic cells. This scheme would allow for mixed tumours. Similarly, arguments can be made for differentiation from centroblasts in follicle centre cell lymphoma. The heterogeneity apparent within NHL and B-cell leukaemia would depend therefore on the differentiation potential of the clonogenic cells as well as the B-cell subset in which it originated.

*G. Janossy* (*London*) presented additional information on the heterogeneity of B-cell populations in the lymphoid tissues. In particular his group have examined foetal tissues where development can occur without the complication of antigenic stimulation. Foetal liver and bone marrow (BM) are important haemopoietic organs; between 20% and 40% of the cells present being of the lymphoid series. Of these, ~30% are immature types which lack surface IgM but have cytoplasmic $\mu$ chains and are TDT+. The remainder have a more mature phenotype (TDT$^-$, BA1$^+$, surface, $\mu^+$, $\delta^-$). A few intermediate forms are also present. Additional B-cell antigens (Y29/55, To15, RFB-4) are missing from the TDT$^+$ cells but appear later as stable markers of peripheral B-lymphocytes. In intermediate cells antigens defined by these antibodies may be seen in the cell cytoplasm. With regard to the peripheral lymphoid organs, by 22 weeks primary follicles have formed with clusters of B-cells (RF-2$^+$ RF B-4$^+$, $\mu^+$, $\delta^-$ Leu-1$^+$) surrounding follicular dendritic cells (DRC), defined with appropriate monoclonal antibodies. The DRC do not at this time have bound complexes. Primary follicle formation is therefore seen to be an event independent of antigen exposure.

The formation of classical germinal centres, is an antigen dependent process. The B-cells present in these early follicles resemble B-CLL cells, and are not represented in BM. Tissue cultures in the presence of phorbol ester and cells of similar phenotype extracted from normal tissues suggest that these cells pass through a stage which resembles hairy cell leukaemia on route to becoming follicle mantle cells. Tartrate resistant acid phosphatase is induced and in normal cells the Leu-1 marker is lost in favour of FMC7 and RFA-4. (Neoplastic cells differ with regard to Leu-1). Information of this type relating to normal cells is of great value in our understanding of the nature and behaviour of tumour cell populations. To conclude the presentation simple protocols for the characterisation of tumour cell populations employing the Fluorescent Activated Cell Sorter were described.

*J. Habeshaw* (*London*) has evaluated the prognostic value of staining cells from non-Hodgkin's lymphoma (NHL) biopsy specimens for transferrin receptor (trf) identified by the OKT9 antibody. Trf is present on a mean of 5% of reactive lymph node cells, with an upper limit of 15%. In lymphomas regarded as being of high grade malignancy the percentage of OKT9$^+$ cells is generally higher with low grade lymphoma forming an intermediate group. Within the high grade lymphomas survival is inversely related to the percentage of OKT9$^+$ cells present. The situation in low grade NHL is less clear, but within this group lymphocytic tumours generally exhibit a lower trf level than lymphomas of follicle centre cell origin. It would appear, therefore, that trf level can be used in combination with histological grading to identify lymphoma patients with a poor prognosis. Animal models demonstrate that trf can be induced on the surface of B-cells by IL-2, a product of activated T-cells. Preliminary studies suggest that in the human lymphoma series studied, biopsies with high OKT9 positivity contain a high percentage of T-cells bearing an activation marker.

The controversial question of histiocytic lymphoma was addressed by *P. Isaacson* (*London*). Unlike the B-cell lymphomas, tumours of true macrophage origin are poorly identified and understood. One reason for this is the difficulty in obtaining histiocytes from tissue biopsies. A major problem concerns the phenotypic markers available. Methods of analysis involving surface receptors together with most of the currently available monoclonal reagents identify blood monocytes and reactive macrophages in preference to tissue histiocytes, from which most lymphomas are derived. The situation is further complicated by the range of cells encompassed within the "macrophage-monocyte series" which include various types of dendritic cell (antigen presenting cells), epithelioid cells and other reticuloendothelial types. This heterogeneity is compounded by the fact that individual macrophage populations change their phenotypic characteristics following activation. It is clear that with careful immunohistochemical techniques using conventional antisera to components of the macrophage cytoplasm (alpha 1 anti trypsin, alpha 1 anti chrymotrypsin and rarely lysozyme) together with certain specific antisera (some developed in Southampton) permit the identification and characterisation of a group of true histiocytic lymphomas. Two cases presented, however, showed that results obtained may occasionally be contradictory and that a full appreciation of the lymphomas of this lineage must await the development of superior markers and improved understanding of the normal cell lineage.

The origin of the Reed Sternberg (RS) cell remains an area of controversy. Previous hypotheses have considered this cell to be derived from lymphocytes, histiocytes or antigen presenting (dendritic) cells, *H. Stein* (*Berlin*) began his discussion of this topic with a definition of Hodgkins disease (HD) which stressed the importance of the correct tissue background as well as the presence of RS and Hodgkins cells (HC) in the diagnosis of this condition. Careful analysis of RS and HC populations in frozen tissue with a range of monoclonal antibodies shows that they cannot be placed within any known cell lineage. The recent development in Germany and Southampton of cell lines of neoplastic karyotype from HD has shed new light on the RS/HC lineage. The cultured cell lines have a phenotype which corresponds closely to the pattern of staining seen on RS and HC in frozen sections. One particular monoclonal antibody (Ki-1) raised to the surface of the L428 cell line stains RS and HC cells, other HD cell lines and a unique population of cells found in the perifollicular area of lymph nodes and tonsils. This cell may be the normal counterpart of RS and HC cells.

Supernatants of the L428 cell line have been shown to exhibit lymphokine-like activity an observation relevant to the development of the reactive stroma in HD. The Southampton cell line, which resembles L428 and carries the Ki-1 antigen, has also been shown to exhibit an isoenzyme pattern for non-specific esterase which is uncharacteristic of any other cell type examined, (*D.B. Jones, Southampton and S. Scott, Leeds*). This result further suggests that RS and HC cells represent a unique cell lineage.

*P. Beverley* (*London*) provided cautionary data on the use of monoclonal antibodies in defining T-cells which complemented earlier comments on cells of the macrophage lineage. It is clear from studies of normal cell populations that phenotype may vary both through development and after neoplastic transformation. The physiological environment in which a cell exists may also affect phenotype. A cloned T-cell line responsive to P20, an antigen derived from influenza virus haemagglutinin, shows increased levels of an activation marker after binding of P20. The level of T3 antigen expression (T-cell receptor associated antigen) is reduced however. In other cases the marker is expressed but not functional.

It is also clear that even with monoclonal antibodies suprising cross reactivities can occur. The antibody UCHT1, raised to peripheral blood lymphocytes, is a valuable reagent for the identification of benign or neoplastic T-cells in biopsy tissue. In the brain Purkinje cells and their processes stain heavily with this antibody. The molecule recognised by UCHT1 in Purkinje cells is probably different from that recognised on T-cells. Within the lymphoid system antibodies which identify the inducer subset in suspension often stain the cytoplasm of macrophages. These observations indicate that monoclonal antibody data on pathological specimens should be interpreted with care and always with regard to the behaviour of normal cell populations.

Idiotype represents one of the most specific of known differentiation antigens. Attack on the tumour idiotype should result in specific damage to the neoplastic population with minimum injury to the normal lymphoid cells. *G.T. Stevenson* (*Southampton*) outlined his experience with anti idiotype therapy. At present certain limitations to effective therapy exist. Exported idiotypic immunoglobulin may produce a barrier which prevents antibody binding to tumour cells and the expression of idiotypic determinants may be modulated by bound antibody. Studies *in vitro* and in the $L_2C$ guinea pig leukaemia model show that univalent antibody derivatives offer promise in avoiding antigenic modulation. These may be constructed in various ways but have in common the retention of the immunoglobulin Fc region. An additional problem is that as yet we do not understand the relative usefulness of the various effector mechanisms which may be initiated by bound idiotype.

The diagnosis of bone marrow involvement is an important part of the staging of lymphoproliferative disease. *F. Pizzolo* (*Verona*) described an evaluation of immunohistochemical techniques in identifying lymphoma cells in bone marrow. Morphological analysis of bone marrow aspirates or trephine biopsies are relatively insensitive as methods of detecting neoplastic cells. Immunohistochemical techniques have improved on both these methods. Despite certain technical problems associated with frozen trephine material, e.g., the presence of fat, bony trabeculae and peroxidase rich haemopoietic elements, it is now possible to routinely stain sections of marrow trephines with a panel of monoclonal and conventional antibodies. Difficulties may be experienced in the definition of monoclonal populations using antibodies to immunoglobulin. Double staining using reagents labelled with two fluorochromes may be useful in the precise identification of unusual tumour cells and minority populations can also be readily identified with appropriate antibodies in marrow infiltrates. The implications of these techniques for our wider understanding of lymphoproliferative disease were shown in a study of B-CLL. Immunohistochemical methods clearly identify the pattern of involvement with neoplastic cells as either nodular or diffuse and further show that the phenotype of the CLL cells is constant in blood and tissue. However, infiltrating T-cells present alongside the neoplastic population differ between blood and marrow. T-lymphocytes of inducer phenotype predominate in the marrow; the reverse of the situation in blood. This observation may be of pathophysiological significance.

Data on marrow involvement were also presented by *J. Smith* (*Southampton*) who described the results of a study of blood and marrow involvement in Non-Hodgkin's Lymphoma (NHL) patients in the Medical Oncology Trial Group. A total of 184 NHL patients have been assessed by the Lymphoma Study Group and phenotypic analysis of blood and marrow is available for 107 of these. It is clear that immunological methods greatly improve the sensitivity of diagnosis of marrow involvement when compared with either cytological analysis of marrow aspirates (53% positive in comparison with 18%) or with the histological examination of trephine biopsies (33%). Monoclonal B-cell populations can also be readily identified in the blood in NHL of low and high-grade malignancy. The analysis of survival data for patients with high grade malignancy shows that the immunological investigation of the bone marrow provides information of value in staging and for prognostic purposes.

In summary phenotypic analysis of tumour cells is of value in the diagnosis and staging of lymphoproliferative disease. Comparison with the behaviour of the appropriate normal populations can give important clues to the behaviour of tumour cells and extranodal involvement can be readily identified. The identification and localisation of accessory cell populations is important in this respect. Problems still exist however. Firstly, we do not yet know enough about the differentiation and behaviour of many of the normal lymphoid cells to make clear statements about origin and behaviour in some lymphomas. The relevance of some of the markers defined by monoclonal antibodies is not yet clear and the data on tumours must be interpreted with caution. Finally, in some lineages appropriate markers do not yet exist to define tumour populations.

The organiser would like to thank the Cancer Research Campaign and the British Association of Cancer Research for financial support and to acknowledge the Session Chairmen, Prof. G.T. Stevenson, Prof. D.H. Wright and Dr. R.L. Souhami. Many members of the University Departments of Pathology and Immunochemistry and the Regional Immunology Service helped with the organisation of the Workshop. The assistance of Mr. B. Mepham and Miss J. Foster is particularly acknowledged.